# ATPase Inhibitory Factor 1—A Novel Marker of Cellular Fitness and Exercise Capacity?

**DOI:** 10.3390/ijms232315303

**Published:** 2022-12-04

**Authors:** Remigiusz Domin, Michał Pytka, Jan Niziński, Mikołaj Żołyński, Ariadna Zybek-Kocik, Elżbieta Wrotkowska, Jacek Zieliński, Przemysław Guzik, Marek Ruchała

**Affiliations:** 1 Department of Endocrinology, Metabolism and Internal Medicine, Poznan University of Medical Sciences, 60-355 Poznan, Poland; 2 University Centre for Sport and Medical Studies, Poznan University of Medical Sciences, 60-802 Poznan, Poland; 3 Department of Cardiology, Intensive Therapy, Poznan University of Medical Sciences, 60-355 Poznan, Poland; 4 Department of Athletics, Strength and Conditioning, Poznan University of Physical Education, 61-871 Poznan, Poland

**Keywords:** inhibitory factor 1, IF1, myokine, exercise-induced cytokine, exercise capacity

## Abstract

ATPase inhibitory factor 1 is a myokine inhibiting the hydrolytic activity of mitochondrial adenosine triphosphate synthase and ecto-F1-ATPase on the surface of many cells. IF1 affects ATP metabolism in mitochondria and the extracellular space and upregulates glucose uptake in myocytes; these processes are essential in physical activity. It is unknown whether the IF1 serum concentration is associated with exercise capacity. This study explored the association between resting IF1 serum concentration and exercise capacity indices in healthy people. IF1 serum concentration was measured in samples collected at rest in 97 healthy amateur cyclists. Exercise capacity was assessed on a bike ergometer at the successive stages of the progressive cardiopulmonary exercise test (CPET). IF1 serum concentration was negatively and significantly correlated with oxygen consumption, oxygen pulse, and load at various CPET stages. A better exercise capacity was associated with lower circulating IF1. IF1 may reflect better cellular/mitochondrial energetic fitness, but there is uncertainty regarding how IF1 is released into the intravascular space. We speculate that lower IF1 concentration may reflect a better cellular/mitochondrial integrity, as this protein is bound more strongly with ATPases in mitochondria and cellular surfaces in people with higher exercise capacity.

## 1. Introduction

Myokines are exercise-induced cytokines with various mechanisms of action. Generally speaking, myokines regulate metabolism, energetic homeostasis, and the structure of cells, tissues, and organs in response to physical activity. Different types of physical effort, i.e., acute or repeated work/exercise and endurance or resistance training, affect most systems and organs in the human body, influence genes encoding myokines, and translate to the myokines’ production and their release into the bloodstream [1,2,3,4,5,6,7]. For example, interleukin-6, the first discovered myokine, increases glucose uptake, induces lipolysis, and modulates muscle growth [8,9,10].

So far, many myokines have been discovered, e.g., myonectin, MG53, myostatin, irisin, follistatin, brain-derived neurotrophic factor, or decorin. Their biological action and metabolic function may overlap, be complementary, or be agonistic [11,12,13,14].

ATPase inhibitory factor 1 (IF1) was discovered by Pullman and Monroy in 1963. IF1 impacts energetic homeostasis by directly regulating mitochondrial ATPase (also called ATP synthase or F0-F1-ATPase) in the oxidative chain and inhibiting the hydrolytic activity of ecto-F1-ATPase on the cell surface (catalytic subunit of ATPase) [15,16]. Recently, Lee et al. described the myokine-like properties of IF1. Along with regulating mitochondrial ATPase, IF1 also influences glucose metabolism by improving glucose tolerance and uptake in myocytes via triggering GLUT4 translocation [17]. These IF1 actions might contribute to better exercise capacity.

Adenosine triphosphate (ATP) is an organic molecule providing energy to many biological reactions in vivid cells, e.g., for contracting muscles. Exercise capacity during endurance sports depends on ATP derived from aerobic metabolism in mitochondria or other energy sources. Repeated endurance training increases mitochondria number, shape, and function and improves energy handling at different physical activity levels. Better exercise capacity usually translates to higher muscle oxygen consumption (VO_2_) in endurance athletes, a faster pace in runners, or a higher achieved load in cyclists.

Although IF1 is involved in energy generation through ATP and glucose metabolism, it is unknown whether the IF1 serum concentration is associated with exercise capacity indices in endurance sports. Such an issue has not been investigated so far. This study aimed to explore the potential correlations between resting IF1 serum concentration and exercise capacity indices measured during a progressive cardiopulmonary exercise test (CPET) on a bike ergometer with healthy amateur cyclists.

## 2. Results

### 2.1. Baseline Characteristics of Studied Participants

The summary of the baseline characteristics and the CPET parameters at all stages is presented in Table 1.

### 2.2. Association of IF1 with CPET Parameters

IF1 concentration was negatively correlated with VO_2_, VCO_2_, O_2_pulse, power/load, and peak exercise (Table 2).

Correlations between IF1 and the remaining CPET parameters were not significant.

## 3. Discussion

We found that resting IF1 concentration was consistently and negatively correlated with exercise capacity indices at various stages of incremental CPET to exhaustion on a cycle ergometer. Healthy amateur cyclists with lower IF1 concentration at rest showed higher VO_2_, VCO_2_, O_2_pulse, and load measured at the warm-up stage, both ventilatory thresholds, and the peak of exercise.

### 3.1. IF1

Muscle contraction and performance depend on ATP content and generation efficiency, both of which rely on oxygen and glucose supply. ATP turnover is regulated by IF1, which is a small protein synthesized in the cytosol, imported to mitochondria, and probably exported to the cell’s surface/extracellular space [16,18,19].

In mitochondria, IF1, depending on its phosphorylation status, binds to ATPase in the last complex of the mitochondrial oxidative chain. On the cell’s surface, IF1 binds to the ectopic ATPase (ecto-F1-ATPase). Ecto-F1-ATPase mainly produces adenosine diphosphate (ADP) from ATP hydrolysis. However, in acidosis or hypoxia, ecto-F1-ATPase may also synthesize ATP [16,20].

### 3.2. IF1 Origin

Although Genoux et al. identified IF1 circulating in human blood in 2011, the origin of this protein is unclear [21]. According to Lee et al., IF1 is secreted by contracting muscles, and its serum concentration increases 1 h after an acute exercise bout [17]. Gore et al. speculated that IF1 is a part of muscle secretome—proteins secreted by contracting muscles and regulating various physio- and pathophysiological processes [16,22]. Gore et al. also alternatively proposed that serum IF1 could be of mitochondrial origin. During ischemia/hypoxia, mitochondria could recruit IF1 from the extracellular space to protect against complete intracellular ATP depletion. Consequently, IF1 could be released back into extracellular space after cells and mitochondria return to normoxic conditions [16]. This hypothesis, however, implies that IF1 is of extracellular and not mitochondrial origin.

### 3.3. Intracellular ATP, ATPase, and IF1

ATP is produced during extra- and intracellular processes, including cytoplasmatic anaerobic glycolysis and mitochondrial oxidative phosphorylation. All ATP synthesized in mitochondria comes from adding a phosphate group to ADP and capturing energy from the proton gradient generated in the electron transport chain. Only ATP synthase synthesizes new ATP molecules in this process [23].

Additionally, mitochondrial ATPase may hydrolyze ATP to ADP during metabolic stress such as hypoxia/anoxia and acidosis [24]. IF1 inhibits the hydrolase activity of ATPase in mitochondria to protect against complete ATP depletion during prolonged metabolic challenges. In this way, IF1 stops the initiation of irreversible apoptosis, leading to cellular death. When the O_2_ supply to cells and mitochondria declines, dephosphorylated IF1 binds with ATPase and stops its hydrolyzing activity [25,26]. The phosphorylated IF1 does not bind with ATPase [27]. Based on these observations, we made a concept explaining lower IF1 serum concentrations in subjects with better exercise capacity in Section 3.6.

### 3.4. Extracellular ATP

Extracellular ATP and its metabolites (ADP, AMP, and adenosine) serve as an energetic supply and signaling molecules in the purinergic system. IF1 regulating the activity of ecto-F1-ATPase affects extracellular and intravascular levels of ATP. Almost all cells release ATP into extracellular space. Nevertheless, ATP’s primary intravenous source remains elusive [28].

ATP in the intravascular space most likely originates from erythrocytes and endothelium. Erythrocytes, however, have the majority of ATP and release it in response to acidosis, hypoxia, beta-adrenergic stimulation, and mechanical deformation. These conditions are present during more intense exercise when muscles or the heart increase the rate and strength of their contractions.

Most of the ATP is released from erythrocytes when they pass the microcirculation of contracting muscles [29]. When ATP gets into the intravascular space, it vasodilates local microcirculation, improves local blood flow during exercise, and keeps that blood flow increased during postexercise recovery. In this way, working muscles are supplied with oxygen and nutrients, while exercise by-products such as CO_2_, lactate, H^+^, and heat are removed. Ectonucleotidases in erythrocytes, endothelium, and other cells hydrolyze an excess of ATP.

Kirby et al. and Mortensen and Saltin suggested that ATP may also be released by contracting skeletal muscles, endothelium, and sympathetic nerve endings, which accumulate in interstitial space. ATP has multidirectional paracrine activity and interacts locally with surrounding cells by purinergic P2 receptors. Interstitial ATP facilitates vasodilation via the endothelium, as well as by the modulation of sympathetic activity. ATP from the interstitial pool will likely not pass to the intravenous space due to the activity of ectonucleotidases instantly hydrolyzing it [30,31,32].

During exercise, free ATP remains stable or increases in the circulating blood [31,33,34]. In highly-trained athletes, resting ATP concentration depends on the training phase during the annual or seasonal cycle, increasing in the peak performance phase [35]. The vasodilating action of ATP in the circulation is mediated through purinergic receptors on endothelial cells and the stimulation of nitrogen oxide (NO) production. [36,37] Referring to these data, we propose a concept explaining lower IF1 serum concentrations in subjects with better exercise capacity in Section 3.7.

### 3.5. Exercise Capacity and IF1

Regular training leads to physiological adaptations in the cardiovascular, respiratory, and musculoskeletal systems and cellular metabolism, thus improving exercise capacity [38]. Mitochondria also adapt to physical exercise and training. Endurance training improves skeletal muscle oxidative capacity by increasing the size and number of mitochondria, the concentration of the Krebs cycle enzymes, the activity of the electron transport chain, and the malate–aspartate shuttle. Consequently, the muscle capacity for aerobic ATP resynthesis rises during exercise [39]. In untrained people, lower oxidative potential shifts ATP production toward anaerobic glycolysis, thus leading to early lactate accumulation and worse exercise performance [40].

VO_2_peak is a marker of exercise capacity in healthy people and patients with various diseases. VO_2_peak reflects the function and interaction among the cardiovascular and respiratory systems, the efficiency of muscle oxygen consumption, and mitochondrial function [41,42]. After VO_2_ is normalized to HR, the O_2_pulse becomes a noninvasive proxy of stroke volume, indirectly reflecting myocardial contractility [43,44]. An individually-attained power depends on muscle function and energy metabolism, including oxygen consumption [40,45,46,47,48,49].

### 3.6. IF1 Is a Potential Marker of Mitochondrial Fitness, Cellular Integrity, and Exercise Capacity

We speculate that lower resting serum IF1 concentration in amateur cyclists with better exercise capacity might reflect more efficient oxygen skeletal muscle supply/utilization and mitochondrial fitness. Supporting this speculation are the significant correlations between IF1 and endurance exercise capacity indices such as VO_2_, O_2_pulse, and power measured during the VT1, VT2, and peak exercise.

**Figure 1:**
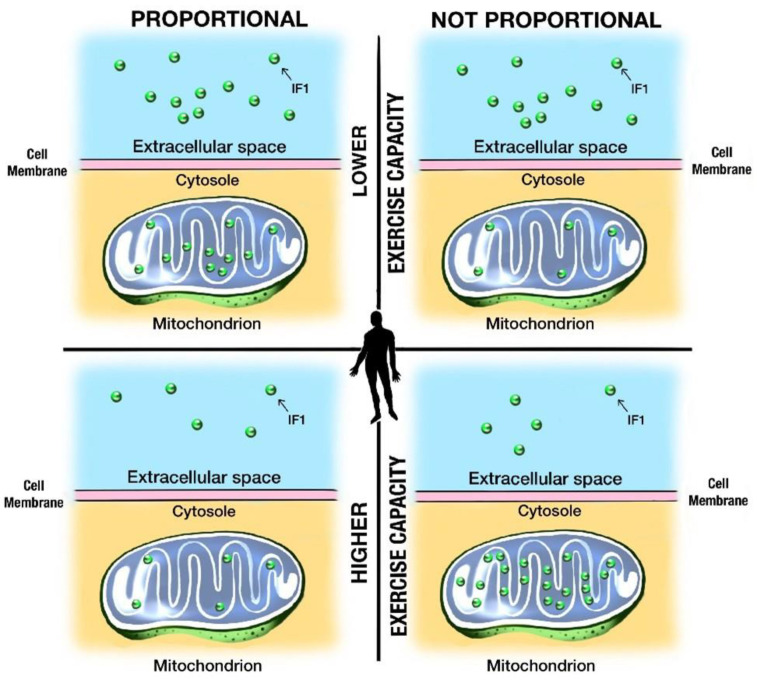
The potential effect of physical activity on mitochondrial membrane integrity and IF1-permeability in two scenarios. Firstly, IF1 in the blood is proportional (left part of the figure) to the IF1 in mitochondria. Secondly, IF1 in the blood is not proportional (right part of the figure) to the IF1 in mitochondria. In the first scenario, non-exercising people with lower exercise capacity are shown in the upper left corner. They need more IF1 to reduce the risk of ATP hydrolysis (and depletion) in mitochondria during occasional physical effort accompanied by hypoxia and acidosis. More IF1 in mitochondria, according to the proportional concept, translates to more IF1 in the blood. In the same scenario, physically active people with better exercise capacity are presented in the bottom left corner. Such individuals do not need a lot of IF1 in mitochondria due to certain other metabolic adaptations protecting against frequent hypoxia/academia bouts during repeated training. Therefore, in the proportional concept, IF1 is either lower or higher in mitochondria and the circulating blood. The second scenario might be explained by not proportional IF1 content in the blood and mitochondria. In this case, lower IF1 in the circulating blood would be associated with a higher mitochondrial IF1 content and vice versa. Non-exercising people with lower exercise capacity (upper right corner) have more IF1 in the blood, as it is not highly demanded in mitochondria, which are not under repeated hypoxia and acidosis. IF1 would be leaked or effluxed from mitochondria to the extracellular and intravascular spaces in such a case. In contrast, the mitochondria of regularly exercising people and those with a better exercise capacity (bottom right corner) need more IF1, so they develop mechanisms to protect IF1 inside and prevent ATP hydrolysis due to more frequent exercise-triggered hypoxia/acidosis. In our opinion, this scenario is more likely and favors the mitochondrial/cell integrity hypothesis. Regular physical activity promotes adaptations to exercise, such as mitochondrial biogenesis, integrity, and the ability of mitochondria to prevent oxidative damage.

The current exercise capacity is a function of training and mitochondrial status. Repeated and more intensive training in people with better exercise capacity leads to several functional adaptations to protect against repeated anaerobic metabolism and acidosis [38]. As we show, cyclists with better exercise performance (as an assumed effect of better/more intensive training) have lower IF1 concentration. How might this finding relate to the mitochondrial IF1 amount? Let us consider two scenarios (Figure 1) regarding IF1 blood concentration and its amount in mitochondria.

First, let us assume that blood IF1 concentration is proportional to the amount of mitochondrial IF1. More IF1 in mitochondria would be reflected by more IF1 in the extracellular and intravascular spaces. If so, less IF1 in the intravascular space might suggest that mitochondria have less IF1, as their ATPase hydrolase activity does not need to be inhibited. However, how likely is this possibility? Athletes who train more frequently develop acidosis and relative cellular hypoxia. Therefore, they might be put at an increased risk of mitochondrial ATPase hydrolyzing ATP when ATP is most needed. It seems, however, that IF1 is in high demand in the mitochondria of people who train more. Therefore, the hypothesis that IF1 in the circulating blood is proportional to mitochondrial IF1 content is less likely.

If the above is so, why do people with better exercise capacity have less IF1 in the circulating blood? Another scenario is plausible: Let us assume that IF1 in the plasma is not proportional to the IF1 content in the mitochondria. More frequent anaerobic metabolism accompanying repeated training suggests that the mitochondria of myocytes should have more IF1 inside than outside the cells, secondary to some mechanisms keeping it inside. Whatever such mechanisms are, they indicate a better cellular/mitochondrial biological condition and integration with less IF1-permeable membranes or better-developed mechanisms protecting against IF1 efflux to the extracellular space. In other words, low IF1 plasma concentration might be a potential marker of mitochondrial and cellular integration.

Individuals with better exercise performance are more often exposed to metabolic disruptions such as relative tissue hypoxia and metabolic acidosis, which might have fatal consequences. Fortunately, nothing like this happens, and physically active people do better than those with sedentary lives. Physical activity and regular exercise have been demonstrated to enhance health and longevity, promote healthy aging, and extend the average life expectancy [50,51]. Exercise protects against mitochondrial dysfunction, impaired mitochondrial quality control, and systemic inflammation at a cellular level. It also promotes mitochondrial biogenesis, integrity, and the ability of mitochondria to prevent oxidative damage [38,51,52,53,54,55,56].

The mitochondria and cells of regularly training or physically working people with better exercise capacity are in better condition. Their membranes are probably more integral—and thus better protected—against IF1 transfer/efflux to extracellular space. This theoretical mechanism might also help to explain why people with a better exercise capacity have lower IF1 blood concentration (Figure 1).

### 3.7. IF1 in Relation to ecto-F1-ATPase, Purinergic Signaling, and Exercise Capacity

There are two other possible hypotheses: vascular and metabolic. IF1 in the blood might not reflect its mitochondrial content for different reasons. These hypotheses would strengthen the concept of cellular/mitochondrial integrity. In such a case, the blood pool of IF1 would be related only to the ecto-F1-ATPase. This might also imply that in people with better exercise performance, IF1 connects with ecto-F1-ATPase more strongly than it does in other individuals. Consequently, IF1 in such cases would be lower in the circulating blood.

The vascular hypothesis is that IF1 circulating in the blood inhibits the hydrolytic activity of the ecto-F1-ATPase of endothelial cells, contributing to the increased pool of intravascular ATP. ATP directly stimulates the P2 family of purinergic receptors in endothelial cells, causing local vasodilation [28]. Proper blood perfusion of muscles is critical for performing physical work [57]. In that case, this increased pool of intravascular ATP could promote vasodilation, translating to better exercise capacity. The second—metabolic—hypothesis is based on the findings of Lee et al., who discovered that C2C12 myotube cells treated with IF1 present a transient increase in extracellular ATP, possibly as the effect of interaction between IF1 and ecto-F1-ATPase on the cell surface. In essence, this causes translocation of the glucose transporter type 4 (GLUT4) to the cell surface and increases cellular glucose uptake [17]. Both acute bouts and regular exercise increase glucose transporter type 4 (GLUT4) expression in myocytes as an adaptation to exercise training [58].

**Figure 2:**
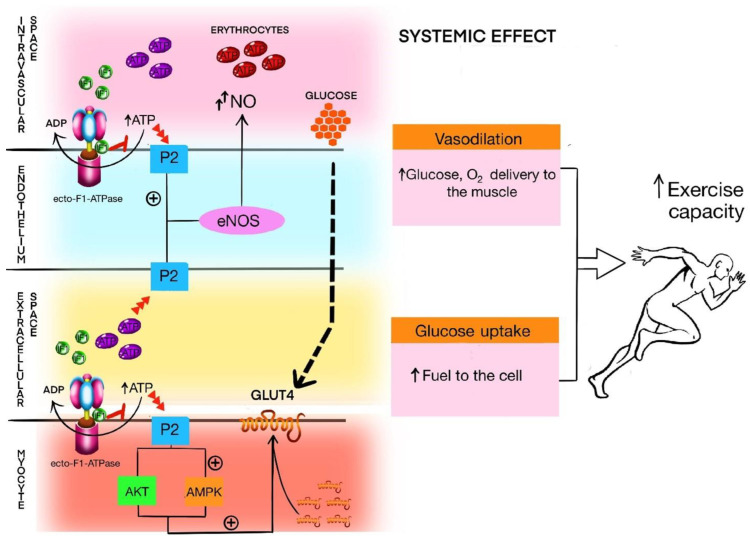
Potential modulation of purinergic signaling by IF1. In intravascular and extracellular space, IF1 binds to ecto-F1-ATPase and inhibits its hydrolytic activity, increasing the pool of circulating ATP. This ATP acts with purinergic P2 family receptors, which, in endothelial cells, activate nitric oxide synthase (eNOS) and nitric oxide (NO) production, thus leading to vasodilation. In muscle cells, an increased pool of extracellular ATP could activate purinergic receptors outside the cell membrane, in turn activating Akt and AMPK kinases. When activated, kinases, AMPK and Akt trigger GLUT4 translocation to the cell surface, thus increasing glucose uptake. Due to vasodilation, the blood delivers more glucose and oxygen to the working muscle. Increased glucose uptake sustains proper glucose delivery to the cell during exercise. Both vasodilation and increased glucose uptake contribute to increased exercise capacity/performance.

Taking these two hypotheses together, we propose that in subjects with better exercise capacity, more IF1 binds to ecto-F1-ATPase to facilitate the increase in intravascular ATP during exercise, and thus to maintain sufficient glucose uptake in muscle cells during exercise. Simultaneously, an increase in ATP, through its direct vasodilatory effects, improves blood flow and O_2_ with nutrients supplied to the muscles. Figure 2 presents the potential modulation of purinergic signaling by IF1.

## 4. Materials and Methods

### 4.1. Bioethical Issues

The Bioethics Committee of the Poznan University of Medical Sciences approved the project (decision number 136/21). The project was conducted following the Declaration of Helsinki [59]. All data were collected confidentially, managed, and stored in the Redcap data capture tool hosted at Poznan University of Medical Sciences (https://redcap.ump.edu.pl). All data were treated anonymously during data storing and analysis. Informed written consent was obtained from all patients before enrollment.

### 4.2. Participants

We enrolled 97 physically active, healthy volunteers of Caucasian race (73 men and 24 woman). The inclusion criterion was at least 1 h of regular exercise per week, while the exclusion criteria were as follows: any chronic disease according to past medical history and regular use of substances from The World Anti-Doping Agency (WADA) list [60].

Participants refrained from vigorous exercise and alcohol for at least 24 h and caffeinated beverages/supplements for at least 12 h before the test. They were asked to come to the laboratory in the postprandial state (~2 h after their last meal).

Each participant signed a written informed consent sheet to participate in the study and was examined by a physician. Blood pressure (BP) and resting heart rate (HR) were assessed at rest (Omron M7 Intelli IT, Omron, Kyoto, Japan, and Polar H10 chest strap heart rate monitor, Polar, Kempele, Finland). Resting BP did not exceed 140/90 mmHg in any participant. Anthropometric parameters were measured using height and an electronic weight scale. After examination and positive qualification for the study, the protocol was continued. Every participant filled in the International Physical Activity Questionnaire (IPAQ) [61].

### 4.3. Pretest Resting Spirometry

Pretest resting spirometry (Vyntus CPX, Vyaire Medical, IL, USA) was performed to measure the forced expired volume in one second (FEV1) and to estimate the maximal voluntary ventilation (MVV) using the formula FEV1 * 40, which is necessary for defining the limit of breathing reserve (BR) during CPET [62].

### 4.4. Cardiopulmonary Exercise Test

CPET was performed on a specialized cycle ergometer (Excalibur Sport 2,Lode, Groningen, The Netherlands) using a CPET system (Vyntus CPX, Vyaire Medical, IL, USA) with gas analyzers determining breath-by-breath oxygen (O_2_) and carbon dioxide (CO_2_) concentrations in inspired and expired air. The CPET system was calibrated following the manufacturer’s recommendations before each test.

CPET was performed to exhaustion according to the Association for Respiratory Technology & Physiology Guidelines (ARTP Guidelines) [62] using an individualized incremental ramp protocol based on participants’ regularity and intensity of training and estimated maximal load, as determined by the Wasserman equation: W = (predicted VO_2_max − VO_2_unloaded)/103 [63]. With this approach, we intended to complete the proper progressive (or incremental) exercise phase of the CPET optimally between 8 and 12 min. Each CPET began with resting recordings, followed by a three-minute warm-up. After the progressive exercise started, it continued until the participants’ exhaustion or until clinically indicated (e.g., due to angina or dyspnea). After subjects stopped pedaling, the registration of recovery started. Heart rate (HR) and CPET parameters were constantly monitored from the rest before the exercise until 15 min of postexercise recovery. The following CPET parameters, either directly measured or derived, were archived discretely for each breath (the breath-by-breath method):

HR—heart rate;
VO_2_—the volume of consumed O_2_ per minute;
O_2_pulse—the ratio of VO_2_ to HR (an indirect measure of stroke volume);
VCO_2_—the volume of produced CO_2_ per minute;
VE—minute ventilation;
TV—the tidal volume of a single breath;
BF—breathing frequency;
VE/VCO_2_—the ventilatory equivalent for O_2_;
VE/VO_2_—the ventilatory equivalent for CO_2_;
RER—respiratory exchange ratio;
PETO_2_—the end-tidal O_2_ tension in the exhaled air;
PETCO_2_—the end-tidal CO_2_ tension in the exhaled air.

The first (VT1) and second (VT2) ventilatory thresholds were determined, using only the incremental exercise phase, by at least two physicians experienced in sports CPET [62]. For the VT1, three methods were used in the following order, i.e., V-slope from the VO_2_ vs. VCO_2_ relationship, VE/VO_2_ (VO_2_ equivalent), and PETO_2_ vs. power. Three other methods were applied for the VT2 measurement, i.e., PETCO_2_ vs. power, VE/VCO_2_ V slope from the VE vs. VCO_2_ plot, and VE/VCO_2_ (VCO_2_ equivalent) [64]. Both VT1 and VT2 thresholds were determined as the best agreement between at least two physicians analyzing the results.

For this investigation, we used averaged CPET values from the last 15 s of the rest and warm-up phases, and preceding VT1, VT2, and peak exercise [65].

### 4.5. Blood Sampling, Storage, and Biochemical Analysis

Peripheral venous blood was collected from the subjects 5 min before the CPET, then centrifuged to obtain serum and stored at −80 degrees Celsius. IF1 serum concentration was estimated with the ELISA method using the Human ATPase inhibitory factor 1 ELISA Kit (Shanghai Sunredbio (SRB) Technology Cat# 201-12-6276, RRID:AB_2924736). According to the assay validation information provided by the manufacturer, sample linear regression with the expected concentration of the correlation coefficient R is higher than 0.95, and the intra-assay variability coefficient is below 9%. Each assay was performed in three replications, and the mean of the results was calculated.

### 4.6. Statistical Analysis

Due to the non-Gaussian data distribution (determined by the D’Agostino-Pearson test), data were summarized as median and the 25th and 75th percentile. We used the nonparametric Spearman correlation with rho coefficient to study associations between IF1 serum concentration and CPET results. Only *p* < 0.05 was considered significant. Statistical analyses were conducted with PQStat Software (PQStat v.1.8.4.124, PQStat, Poznań, Poland).

## 5. Conclusions

### 5.1. Limitations

We measured neither IF1 intracellular nor mitochondrial content. Therefore, whether or not IF1 serum concentration is proportional to the intracellular and mitochondrial IF content has not been determined. However, direct measurements of IF1 in the myocytes and their mitochondria would require an invasive biopsy, which is usually problematic in physically active people. An animal model might be helpful.

Furthermore, we performed this study with healthy amateur cyclists from the Caucasian race, between 19 and 55 years of age. For these reasons, our findings cannot be extrapolated to a broader population, including children/adolescents, people over 55 years old, those from other ethnic groups, and those who undertake sports other than cycling. Additionally, our observations cannot be applied to patients with various diseases.

### 5.2. Perspectives

IF1 resting serum concentrations are negatively correlated with indices of exercise capacity in healthy amateur cyclists at different stages of an incremental CPET. Lower IF1 concentrations are related to better exercise capacity.

Initial data suggest that resting plasma IF1 has the potential to become a biochemical marker for approximating exercise capacity/training status in athletes. If the concept that IF1 in plasma is not proportional to the IF1 content within cells and mitochondria is true, then IF1 might also be a marker of the biological integrity of cells and their mitochondria. Future investigations should address the issues of the limitations of our study by, for example, including broader age groups, athletes training in various sporting disciplines, different clinical scenarios, and possible effects of pharmacological agents and supplements. A summary of current state of knowledge is presented in Box 1.

Box 1 **Knowns, unknowns, and speculations about IF1 and ATP.**

- ATP is crucial in all features of physical activity and skeletal muscle work, including exercise capacity;
- IF1 regulates the activity of mitochondrial ATPase and ecto-F1-ATPase on the cells surface;
- ATPase and ecto-F1-ATPase regulate the amount of ATP and its metabolites in intra- and extracellular space;
- IF1 serum concentration negatively correlates with indices of exercise capacity;
- How IF1 migrates from mitochondria to extracellular space and further to intravenous space is unknown;
- IF1 might be a novel marker of cellular fitness, cellular integrity, and exercise capacity.

## Figures and Tables

**Table 1:** Summary of CPET parameters measured at rest, warm-up, VT1, VT2, and peak exercise.

	Median	LQ	UQ	Median	LQ	UQ	Median	LQ	UQ	Median	LQ	UQ	Median	LQ	UQ
STAGE	Rest			Warm-up			VT1			VT2			Peak Exercise		
Age	29	26	35	-	-	-	-	-	-	-	-	-	-	-	-
BMI [kg/m^2^]	23.67	21.63	25.93	-	-	-	-	-	-	-	-	-	-	-	-
IF1 serum concentration [ng/L]	646	427	1940	-	-	-	-	-	-	-	-	-	-	-	-
BF [breaths/min]	15.44	11.83	18.99	21.33	18.32	25.17	25.97	22.6	29.93	32.94	28.08	37.42	53.05	44.84	63.26
VE/VCO_2_	32.68	30.32	36.09	26.69	25.24	27.81	25.26	23.61	26.9	26.35	24.39	28.08	32.48	29.98	35.67
VE/VO_2_	29.25	26.56	35.29	23.26	20.65	24.6	25.1	23.23	26.64	28.94	26.7	31.13	39.4	37.08	45.22
HR [beats/min]	83	73	95	119	106	128	153	146	162	173	166	182	187	179	192
VE [L/min]	14.82	11.17	18.87	36.95	30.67	43	64.18	52.27	76.8	95.42	78.52	107.01	149.16	122.71	173.03
O_2_pulse [mL/beat]	4.83	3.63	6.22	12.46	10.69	14.77	15.84	13.19	18.55	18.04	15.56	20.76	18.98	16.03	21.37
PETCO_2_ [Pa]	4221.03	3878.64	4454.43	5248.57	5061.57	5489.65	5539.82	5281.43	5886.86	5367.15	5084.8	5789.04	4469.03	4025.27	4818.42
PETO_2_ [Pa]	15,329.75	14,738.12	16,115.79	13,923.84	13,372.9	14,350.91	14,100.66	13,706.29	14,523.81	14,780.66	14,302.99	15,108.75	16,056.14	15,689.28	16,482.93
RER	0.9	0.82	1.05	0.86	0.79	0.92	0.99	0.97	1	1.1	1.08	1.13	1.26	1.22	1.3
VCO_2_ [L]	0.39	0.26	0.53	1.3	1.02	1.51	2.39	1.95	2.84	3.43	2.93	3.97	4.41	3.63	5.02
VO_2_/kg	5.4	4.07	7.15	19.15	17.21	22.78	31.63	27.48	36.57	40.55	34.49	45.78	45.23	38.97	50.38
VO_2_ max [L]	0.42	0.3	0.53	1.49	1.23	1.74	2.42	1.96	2.91	3.09	2.6	3.65	3.43	2.97	3.98
TV [L]	0.84	0.64	1.2	1.67	1.35	2.01	2.48	2	2.95	2.93	2.45	3.36	2.83	2.44	3.16
Load [W]				70	50	80	183.97	139.98	224.93	254	214.99	304	299	260	365
BR (%)	90.89	87.92	92.74	76.93	72.54	80.46	59.1	52.96	66.58	41.87	30.72	48.96	8.16	−6.68	21.05
BR [L]	145.97	120.98	166.46	125.88	98.98	148.68	93.95	74.89	117.25	63.96	45.38	87.82	10.9	−9.34	32.31

BF—breathing frequency; BR—breathing reserve; HR—heart rate; PETCO_2_—end-tidal pressure for carbon dioxide; PETO_2_—end-tidal pressure for oxygen; RER—respiratory exchange ratio; TV—tidal volume; VCO_2_—the volume of exhaled carbon dioxide; VE—minute ventilation; VE/VCO_2_—ventilatory equivalent for carbon dioxide; VE/VO_2_—ventilatory equivalent for oxygen; VO_2_—the volume of consumed oxygen; O_2_pulse—the ratio of VO_2_ to HR.

**Table 2:** Association of IF1 with CPET parameters during different stages of test.

	Warm-Up		VT1		VT2		Peak Exercise	
	r	*p*-Value	r	*p*-Value	r	*p*-Value	r	*p*-Value
VE/VCO_2_	0.07	0.5191	−0.04	0.7323	0	0.9929	0.11	0.2897
VE/VO_2_	0.01	0.888	−0.04	0.7292	0	0.9626	0.09	0.4019
HR	0.03	0.7607	0.01	0.9203	0.04	0.7297	0.14	0.1794
VE	−0.13	0.1947	−0.26	0.0112 *	−0.23	0.0214 *	−0.16	0.1193
O_2_pulse	−0.26	0.0111 *	−0.24	0.0193 *	−0.22	0.0342 *	−0.21	0.0373 *
PETCO_2_	−0.14	0.1833	−0.01	0.9276	−0.04	0.7313	−0.17	0.0957
PETO_2_	0.07	0.4716	−0.01	0.9228	0	0.9646	0.1	0.331
RER	0.03	0.7854	0.01	0.9114	−0.05	0.6564	−0.05	0.6575
VCO_2_	−0.18	0.0814	−0.23	0.0211 *	−0.23	0.0237 *	−0.21	0.0399 *
VO_2_/kg	−0.04	0.728	−0.19	0.0692	−0.15	0.14	−0.12	0.2454
VO_2_	−0.2	0.0473 *	−0.22	0.0288 *	−0.21	0.0354 *	−0.21	0.0431 *
TV	−0.15	0.1317	−0.16	0.1089	−0.15	0.1321	−0.18	0.076
Load	−0.2	0.0526	−0.27	0.0076 *	−0.27	0.0067 *	−0.26	0.0107 *
Breathing reserve (%)	0.1	0.3111	0.18	0.0811	0.15	0.1497	0.08	0.4255
Breathing reserve (L)	−0.04	0.7266	0	0.9983	0.03	0.7432	0.09	0.3774

BF—breathing frequency; BR—breathing reserve; HR—heart rate; PETCO_2_—end-tidal pressure for carbon dioxide; PETO_2_—end-tidal pressure for oxygen; RER—respiratory exchange ratio; TV—tidal volume; VCO_2_—the volume of exhaled carbon dioxide; VE—minute ventilation; VE/VCO_2_—ventilatory equivalent for carbon dioxide; VE/VO_2_—ventilatory equivalent for oxygen; VO_2_—the volume of consumed oxygen; O_2_pulse—the ratio of VO_2_ to HR. * *p* < 0.05.

## Data Availability

The data presented in this study are available on request from the corresponding author.
